# An Observational Study to Evaluate the Effectiveness of Rivaroxaban in the Management of Cerebral Venous Sinus Thrombosis

**DOI:** 10.7759/cureus.13663

**Published:** 2021-03-03

**Authors:** Meraj Fatima, Muhammad Sohaib Asghar, Saira Abbas, Samar Iltaf, Aijaz Ali

**Affiliations:** 1 Neurology, Dow University of Health Sciences, Karachi, PAK; 2 Internal Medicine, Dow University of Health Sciences, Karachi, PAK; 3 Neurology, Jinnah Medical College Hospital, Karachi, PAK

**Keywords:** cerebral venous sinus thrombosis (cvst), rivaroxaban, direct factor xa inhibitors, heparin, warfarin therapy, direct oral anticoagulant therapy, venous thrombotic event, catheter-directed thrombolysis, neuroimaging, magnetic resonance venogram

## Abstract

Background and objectives

Cerebral venous sinus thrombosis (CVST) is a relatively rare and underdiagnosed type of stroke. Rivaroxaban is licensed for venous thromboembolism in patients opting for elective knee and hip replacement surgeries, preventing pulmonary embolism and precluding stroke and systemic embolism in sufferers of non-valvular atrial fibrillation. Beneficial outcomes depicting the efficacious role of rivaroxaban in treating CVST are under study.

Materials and methods

We performed a prospective observational study in patients diagnosed with CVST in the medicine or neurology unit of a tertiary care hospital in Karachi, Pakistan, between January 2019 and December 2019. The diagnosis of CVST was made by magnetic resonance venography (MRV) in all the cases. Follow-up visits were scheduled at three months and six months, and the occurrence of thrombotic events or bleeding complications was recorded. Follow-up was done by magnetic resonance imaging at three and six months to assess vessel recanalization. Excellent outcome was defined as a modified Rankin Scale (mRS) of 0 or 1. A total of 31 patients were meeting the inclusion criteria and were inducted into the study after informed consent.

Results

The mean age of the study population was 35.11 ± 8.96 years with 71% females and 29% males. The most prevalent etiology was the pregnancy/postpartum period (52%) followed by antiphospholipid syndrome (23%). The frequent clinical manifestations were headache (84%) followed by vomiting (38%), altered level of consciousness (35%), focal deficit/limb weakness (32%), aphasia (29%), blurring of vision (26%), and seizures (23%). Radiological studies showed that the vessels chiefly occluded in our study were superior sagittal sinus (29%), transverse sinus (23%), sigmoid sinus (16%), jugular vein (9%), and cortical veins (3%). Common features on magnetic resonance imaging (MRI) were cerebral edema (45%), hemorrhage (39%), infarct incidence (32%), and raised intracranial pressure (26%). Clinical outcomes showed 55% of patients had partial recanalization and 39% had complete recanalization after a period of six months of the administration of rivaroxaban. Ninety-three percent (93%) of sufferers recovered excellently according to mRS and only 3% developed recurrent CVST within a span of six months. The frequency of thrombotic events and bleeding complications were reported in 6% of patients, respectively, while mortality reported was also 6%.

Conclusion

Rivaroxaban has shown promising results in the management of our CVST patients, hence, it further warrants randomized controlled trials of rivaroxaban against conventional treatments to prove its significant role.

## Introduction

Cerebral venous sinus thrombosis (CVST) is a relatively rare and underdiagnosed type of stroke, constituting approximately 0.5% to 1% of all patients of stroke [1]. The prevalence of CVST is calculated in recent times to be 1.32 per 100,000 every year in Western Europe with a peak being observed in developing countries [2]. CVST is prevalent in adolescents, with females being affected more than males. The mean age of a patient affected by CVST is reportedly 39 years, but it can rarely affect the elder population [3-4]. CVST is a consequence of numerous reversible or non-reversible causative factors, including (1) prothrombin mediated like factor V gene mutation, protein C and S deficiency, anti-thrombin III deficiency, and anti-phospholipid antibodies; (2) inflammatory causes like inflammatory bowel diseases, Crohn's, and ulcerative colitis and their remedy with steroids are involved in potentiating the risk of CVST; (3) pregnancy, puerperium, malignancies, and various hyper-clotting entities; (4) sinusitis, trauma, and surgical interventions of the brain; (5) drugs involved in triggering CVST, including oral contraceptive pills (OCPs), tamoxifen, erythropoietin, and heparin [1,3].

CVST manifests with diverse clinical outcomes ranging from mild, including headache affecting 83% of the patient, moderate, including signs of intracranial hypertension such as headache with papilledema and focal deficiencies like paresis and aphasia frequently coupled with seizures, and severe condition attributing to encephalopathy, coma, and status epilepticus [1-3]. Clinically, on the basis of severity, CVST is divided into the acute (24%), sub-acute (64%), and chronic (12%) categories [3]. In patients with CVST, the lateral and superior sagittal sinuses are most commonly affected [3]. Computed tomography (CT) brain is extensively used and easily operated, however, magnetic resonance imaging (MRI) of the brain with T1- and T2-weighted films correlated with magnetic resonance angiography contribute as the gold standard diagnostic investigation for CVST. High spiking levels of D-dimer are constantly present in a sufferer of CVST [3].

Curative modalities opted for CVST constitute heparin, oral anticoagulation with vitamin K antagonist (VKAs) for three to six months [4-5]. However, recently conducted research is pointing toward the utilization of new oral anticoagulants for the remedy of CVST, as they directly target either thrombin or factor Xa [1,3,5]. Dabigatran, rivaroxaban, apixaban, and edoxaban are collectively named as new oral anticoagulants opted for the treatment of cerebral venous sinus thrombosis. Rivaroxaban, a new oral anticoagulant target antagonizing factor Xa, hindering the production of thrombin selectively thus blocking the cascade of intrinsic and extrinsic pathways for coagulation [3,6]. Rivaroxaban, today, is licensed for obviating venous thromboembolism in patients opting for elective knee and hip replacement surgeries, preventing pulmonary embolism, and precluding stroke and systemic embolism in sufferers of non-valvular atrial fibrillation [3,6]. Beneficial outcomes depicting the efficacious role of rivaroxaban in treating cerebral venous sinus thrombosis are attaining and sustaining ideal levels of the international normalization ratio (INR). Rivaroxaban accounts for declining chances of fatal bleeding and intracranial hematoma [2-4,6].

Adverse outcomes associated with rivaroxaban therapy are hepatobiliary disorders, allergic and hypersensitivity reactions, leukocytoclastic vasculitis, and alopecia [7]. Utilization of rivaroxaban and other drugs belonging to the family of non-vitamin K antagonist oral anticoagulants (NOA) are prohibited in sufferers of renal and hepatic ailments, patients with mechanical heart valves, while used cautiously in adolescents and the elder population [3,8-9]. This study aims to find out the effectiveness of rivaroxaban in CVST patients.

## Materials and methods

We performed a prospective observational study in 31 patients diagnosed with CVST in the medicine or neurology unit of a tertiary care hospital in Karachi, Pakistan, between January 2019 and December 2019. All clinical data were extracted from the patient records using a standardized proforma, and informed consent was obtained from all patients on follow-up. The diagnosis of CVT was made by magnetic resonance venography in all the cases. Follow-up visits were scheduled at three months and six months and the occurrence of thrombotic events or bleeding complications was recorded. Follow-up was done by magnetic resonance imaging at three and six months to assess vessel recanalization. Excellent outcome was defined as a modified Rankin Scale (mRS) of 0 or 1 [2]. Comparative to European studies, only three patients were given heparin for initial 5-7 days followed by starting the patient on rivaroxaban (20 mg once daily). The median duration of treatment with rivaroxaban was 24 weeks. The rest 28 patients were given rivaroxaban 20 mg daily for at least six months. No patient received concomitant antiplatelet drugs or any other therapy that can interact with heparin or rivaroxaban in our subjects.

Inclusion criteria included patients who were diagnosed with CVST on radiological studies and were treated with rivaroxaban without dosage skipping or withdrawal of the drug (uninterrupted therapy). The exclusion criteria were a diagnosis of ischemic or hemorrhagic stroke without any venous thrombosis (n=5) and those with interrupted therapy (n=2). A total of 31 patients were meeting the inclusion criteria and were inducted into the study after informed consent. Follow-up radio imaging was assessed after the third and sixth months of rivaroxaban therapy. The outcomes were reported after assembling the data into the Statistical Package for the Social Sciences (SPSS) version 25.0 (IBM Corp. Armonk, NY, USA). The mean age and duration of hospital stay are described as mean and standard deviation. The qualitative data are presented as frequency and relative percentages.

## Results

The mean age of the study population was 35.11 ± 8.96 years with females (71%) being prominent sufferers of CVST than males (29%). The most prevalent etiology was pregnancy/postpartum period (52%), the second common cause was antiphospholipid syndrome (23%) while the least frequent cause was genetic thrombophilia and smoking (3%). The most characteristic clinical manifestation recorded in our study was headache (84%), followed by vomiting (38%), altered level of consciousness (35%), focal deficit/limb weakness (32%), aphasia (29%), blurring of vision (26%), and seizures (23%) while the least encountered manifestation was paresthesia with 9% frequency.

The radio imaging showed vessels chiefly occluded in our study were superior sagittal sinus with involvement in 29% of the cases, succeeded by transverse sinus (23%), the third-most occluded vessel was the sigmoid sinus (16%), the jugular vein was thrombosed in 9% individuals, and the infrequently involved vessels were cortical veins (3%). Common features on magnetic resonance imaging (MRI) were cerebral edema accounting for 45%, followed by cerebral hemorrhage with a 39% incidence, cerebral infarcts with 32% incidence, while insignificant lesions recorded in our study were signs of raised intracranial pressure (ICP) with 26% incidence.

The clinical outcomes showed 55% of patients had partial recanalization and 39% had complete recanalization after a period of six months of administration of rivaroxaban. Ninety-three percent (93%) of sufferers in our study population recovered excellently from CVST according to the mRS, and only 3% of sufferers developed recurrent CVST within a span of six months. The frequency of thrombotic events and bleeding complications were reported in 6% of patients, respectively, while the death ratio reported in our drug trial was also 6%. All the major outcomes and study variables are tabulated in Table 1.

**Table 1:** Baseline data and various outcomes of involved patients in our study (n=31) CVST: cerebral venous sinus thrombosis; ICU: intensive care unit; mRS: modified Rankin scale

Age	
Mean age (years)	35.11 ± 8.96
Gender	
Males	9 (29%)
Females	22 (71%)
Comorbidities	
Diabetes	3 (9%)
Hypertension	5 (16%)
Ischemic heart disease	2 (6%)
Chronic kidney disease	1 (3%)
Autoimmune disease	7 (23%)
History of hospitalization with CVST	
Yes: 28 (90%)	No: 3 (10%)
Mean duration of hospital stay	7.13 ± 3.39
ICU admissions	4 (13%)
Any known cause of CVST	
Pregnancy/Postpartum period	16 (52%)
Antiphospholipid syndrome	7 (23%)
Anemia (or any other hematological deficiency/disease)	2 (6%)
Hereditary thrombophilia	1 (3%)
Head trauma	2 (6%)
Oral contraception	2 (6%)
Smoking	1 (3%)
Clinical presentation	
Headache	26 (84%)
Vomiting	12 (38%)
Seizures	7 (23%)
Focal deficits	10 (32%)
Difficulty in speech	9 (29%)
Visual problems	8 (26%)
Paresthesia	3 (9%)
Altered level of consciousness	11 (35%)
Chief sinus involved	
Superior sagittal sinus	9 (29%)
Transverse sinus	7 (23%)
Sigmoid sinus	5 (16%)
Cortical veins	1 (3%)
Jugular vein	3 (9%)
More than one sinus involved simultaneously	6 (19%)
MRI findings	
Edema	14 (45%)
Hemorrhage	12 (39%)
Venous infarction	10 (32%)
Raised intracranial pressure	8 (26%)
Outcome after 6 months	
Excellent (mRS 0–1)	29 (93%)
Partial recanalization	17 (55%)
Complete recanalization	12 (39%)
Any thrombotic event during study	2 (6%)
Recurrent CVST	1 (3%)
Bleeding (major or minor)	2 (6%)
Death	2 (6%)

## Discussion

Multiple studies regulated in a similar pattern reported a mean age of 36 years (with an age range of 16-70), an outcome correlating with the finding of our study [2,5,10-11]. Gender discrimination was evident within a copious number of studies with the female gender prominently suffering from CVST, a finding corresponding to our results [12-16].

The increased frequency of OCP's use was reported in many studies as a causative factor of CVST among the female gender [2,10,16]. In our study, only 6% of patients reported OCPs as leading causative agents, a finding synchronizing with few studies [5,14]. Our study quoted 3% of patients with smoking as the least frequent cause for CVST, an outcome synchronizing with a limited number of studies reporting similar findings [5,15]. Hematological disorders were encountered among 6% of sufferers engaged with our study, an outcome parallel with a few trials [10]. Hematological disorders were prominent in the Portuguese population [11]. A greater number of studies concluded increased frequency of genetic thrombophilia when compared with 3% reported in our study [2,5]. No genetic thrombophilia was observed in the trial conducted by Marcelo et al. [15]. A specific number of studies quoted the frequency of steroids as a source of encountering CVST [5,11,15]. A negligible number of studies quoted the frequency of previous thromboembolic events causing CVST [5,14]. No occurrence of previous thromboembolic events was recorded in trials conducted within Italian and Portuguese populations [2,15]. An extensive number of studies reported pregnancy/puerperium as the least source of projecting sufferers to CVST, which coincided with the majority of sufferers in our study [5,10,14-16].

Multiple studies reported headache as a prominent manifestation of CVST, with 84% frequency quoted by our study [2,15]. A great number of studies reported an increased frequency of headaches as compared to our study [10,11,14]. Seizures were reported by 23% of inhabitants engaged in our drug trial, an outcome corresponding with various studies quoting similar frequency [2,5,11]. Increased frequency of seizures in CVST patients was observed in a few studies [10,15,16]. Paresis is another prominent manifestation of CVST, increased frequency is reported by multiple studies [10,11,14-15], not correlating with 32% quoted by our study [2,10,11,14-15]. The frequency of papilledema as a manifestation of CVST was recorded in 25% of patients of our study, i.e., having raised ICP, an outcome contrasting with numerous studies [10,11,14]. The frequency of aphasia as a manifestation of CVST in our study was recorded as 29%, a finding correlating with few studies [5,11], increased frequencies were observed in two studies [2,10], and decreased frequency in two other studies [14-15] thus contrasting our results. In our study, 26% of inhabitants suffered visual impairment as a symptom of CVST, an outcome correlating with few studies [5,15] while the increased frequency was recorded in one study [2], as opposed to decreased in others [10,14]. A trivial number of studies quoted isolated intracranial hypertension as the least recorded symptom of CVST with 25% of sufferers recorded in our study [11,16].

Superior sagittal sinus was the most common vessel affected reported by multiple studies along with a frequency of 29% engaged in our trial, an outcome correlating with few studies [5,14]. Increased frequencies were also observed in a handful number of studies [10,15]. The vessel primarily affected in CVST as quoted by numerous study trials is transverse sinus [2,5,11,14-15]. The frequency of transverse sinus being affected was recorded as 23% in our study, much less than previously reported. The jugular sinus is the third most involved vessel in CVST concluded by plenty of studies, and our results showed 9% involvement, an outcome contrasting with them [5,14-15]. Cortical veins and deep venous systems were the least occluded sinuses in miscellaneous studies, similar to our results [5,14-16]. No involvement of the cortical and deep venous system was revealed in another study trial conducted by Anticoli et al [2].

Cerebral hemorrhage was the most prevalent lesion detected in radio imaging within sufferers of CVST with a frequency of 39% in our study, a conclusion not synchronizing with many studies [2,10,15]. Cerebral edema, with a 45% incidence, was the most prevalent lesion detected in neuroimaging outcomes of our study, multiple other studies also followed our results [2,10]. Cerebral infarcts were encountered in 32% of lesions of our study participants, an outcome coinciding with countable studies [2,11]. A study conducted by Marie Girot et al. concluded its increased frequency thus contrasting our outcome [16].

Partial recanalization was observed in 55% of patients engaged in our drug trial, a conclusion correlating with multiple studies [5,15]. The frequency of the complete recanalization was decreased as compared to partial with 39% of individuals achieving it in our study, contrary to a few studies [2,15]. No recanalization was also encountered in a study conducted within the Portuguese population [15]. A limited number of patients also developed recurrent CVST in multiple studies along with 3% reported in our sample population [11,17] while a study regulated by Ferro et al. along with studies conducted within Portuguese and Italian populations concluded no occurrence of recurrent CVST [2,14-15]. Another study conducted in a similar pattern reported the male gender more susceptible to recurrence of CVST [18]. The incidence of complete recovery recorded was 93% in our study, contradicting with outcomes of a few studies [10-11]. Death frequency recorded in participants involved in our drug trial was 6%, coinciding with many studies [10-11,16].

There were a few limitations of the current study. First of all, single-center observations with a limited sample size. There is no control group, lack of randomization, comparable data toward conventional therapies are not available. Hence, it limits the overall projection of therapeutic benefits unless proved by a randomized control trial.

## Conclusions

Conventionally, CVST was treated with either thrombolysis or long-term warfarin therapy. In resistant cases, surgical intervention was needed. Rivaroxaban has shown promising results in the management of CVST in this study cohort. Further randomized controlled trials (RCTs) are needed to prove its significance. It would be interesting to see in future studies if rivaroxaban has any role in preventing surgical intervention against conventional therapies.
